# *In Vitro* Intestinal Permeability Studies and Pharmacokinetic Evaluation of Famotidine Microemulsion for Oral Delivery

**DOI:** 10.1155/2014/452051

**Published:** 2014-12-07

**Authors:** Sajal Kumar Jha, Roopa Karki, Venkatesh Dinnekere Puttegowda, D. Harinarayana

**Affiliations:** ^1^Department of Pharmaceutics, Bengal College of Pharmaceutical Sciences & Research, BRB Sarani, Bidhannagar, Durgapur 713212, India; ^2^Department of Industrial Pharmacy, Acharya & B.M. Reddy College of Pharmacy, Soldevanahalli, Bangalore 560090, India; ^3^Nishka Scientific & Reference Laboratories, Hyderabad, India

## Abstract

The absolute bioavailability of famotidine after oral administration is about 40–45% and absorbance only in the initial part of small intestine may be due to low intestinal permeability. Hence, an olive oil based microemulsion formulation with Tween-80 as surfactant and PEG-400 as cosurfactant was developed by using water titration method with the aim of enhancing the intestinal permeability as well as oral bioavailability.* In vitro *drug permeation in intestine after 8 h for all formulations varied from 30.42% to 78.39% and most of the formulations showed enhanced permeation compared to pure drug (48.92%). Famotidine microemulsion exhibited the higher absorption and *C*_max⁡_ achieved from the optimized famotidine formulation (456.20 ng·h/ml) was higher than the standard (126.80 ng·h/mL). It was found that AUC_0–24_ h obtained from the optimized famotidine test formulation (3023.5 ng·h/mL) was significantly higher than the standard famotidine (1663.3 ng·h/mL). F-1 demonstrated a longer (6 h) *T*_max⁡_ compared with standard drug (2 h) and sustained the release of drug over 24 h. The bioavailability of F-1 formulation was about 1.8-fold higher than that of standard drug. This enhanced bioavailability of famotidine loaded in microemulsion system might be due to increased intestinal permeability.

## 1. Introduction

Peptic ulcer comprises heterogeneous disorders, which manifest as a break in the lining of the gastrointestinal mucosa bathed by acid and pepsin. It is the most predominant of the gastrointestinal diseases with a worldwide prevalence of about 40% in the developed countries and 80% in the developing countries. In recent years large advance in chemical and pharmacological studies has contributed to the knowledge about new therapeutically active compounds and control drug delivery systems for peptic ulcer. Out of the available category of drugs for the treatment of ulcer, H_2_ antagonist's class of drugs like famotidine and ranitidine is considered to be the safest drugs available [1]. Hence, these drugs have promising future if controlled release formulations are made. Famotidine is* N*′-(amino sulfonyl)-3-[[[2-[(diamine methylene) amino]-4-thiazolyl] methyl] thio] propanimidamide a model BCS class-III drug. It is a potent H_2_ receptor antagonist used to treat peptic ulcer and hence effectively heals gastric and duodenal ulcers and is also effective in Zollinger-Ellison Syndrome. The BCS classification provided new quantitative data of importance for modern drug development, especially within the area of drug permeability. It gives clear and easy applied rules for determining the rate limiting factors of GI absorption process. For a BCS class-III drug we need to increase its permeability to improve its oral bioavailability because, here, in class III drugs they have high solubility but low permeability. The absolute bioavailability of famotidine after oral administration is about 40–45% [2] and absorbance only in the initial part of small intestine may be due to low intestinal permeability. Microemulsions as colloidal carriers are one of the promising systems that have nowadays attracted the main interest in intestinal permeability enhancement. They are optically isotropic, transparent, and thermodynamically stable homogeneous solutions of oil and water, stabilized by addition of a surfactant and usually a cosurfactant [3, 4]. Hence, an olive oil based microemulsion formulation with Tween-80 as surfactant and PEG-400 as cosurfactant was developed by using water titration method with an aim of enhancing the intestinal permeability as well as oral bioavailability [5]. The surfactant and cosurfactant (Tween-80 and PEG-400) may have contributed to an increase in the permeability of the intestinal membrane or improved the affinity between lipid particles and the intestinal membrane.

## 2. Materials and Methods

### 2.1. Materials

Famotidine USP was obtained from Micro Labs (Bangalore, India) as free gift sample. PEG-400 was purchased from B.D. Pharmaceuticals Ltd. (Kolkata, India); Tween-80 was purchased from Merck Specialties Pvt. Ltd. (Mumbai, India). HPLC-grade methanol, ammonium acetate, and ethyl acetate were purchased from Sigma-Aldrich, India. All other chemicals used in this study were obtained commercially and were of analytical (AR) grade.

### 2.2. Preparation and Characterization of Formulations

Olive oil based microemulsion formulations were developed (phase titration method) with Tween-80 as surfactant and PEG-400 as cosurfactant, keeping constant weight ratio of surfactant/cosurfactant (2 : 1). Drug loaded microemulsion system (F-1) was prepared by dispersing famotidine (40 mg) into the mixture of surfactant cosurfactant and oil followed by precise addition of water drop by drop to the oily phases with magnetic stirring at ambient temperature. After the resulting systems were equilibrated with gentle magnetic stirring, they were ultrasonicated. Droplet size distribution of optimized microemulsion was determined by using a Delsa Nano-C (Beckman Coulter Instruments) based on light scattering phenomenon, which analyzes the fluctuations in light scattering. Percentage transmittance of samples was measured at 650 and 400 nm with distilled water taken as blank and three replicates were performed for each sample. The pH values of the microemulsion were measured by a pH meter (Digital Systronics, Mumbai, India) at ambient temperature with glass electrode. The viscosity measurement of the prepared microemulsion was performed using Brookfield's viscometer (Brookfield LVDV-II + proviscometer) at single mode using spindle number CPE41 at 32 ± 0.5°C. All aspects of testing were controlled using Rheocalc software.

### 2.3. *In Vitro* Intestinal Permeation Studies

To check the intraduodenal permeability, the duodenal part of the intestine was isolated and taken for the* in vitro* permeation study. Then this tissue was thoroughly washed with phosphate-buffered saline (pH 6.8) solution to remove the mucous and lumen contents. The microemulsion sample of approximately 1 mL was injected into the lumen of the duodenum using syringe, and the two sides of the intestine were tightly closed. The receiver compartment was filled with 45 mL of phosphate-buffered saline (pH 6.8) with continuous aeration and a constant temperature of 37°C. Mixing was performed by means of a magnetic stirrer at 50 rpm, 1 mL samples were withdrawn periodically from the receiver compartment at time intervals of 30 minutes, 1 h, 2 h, 3 h, 4 h, 5 h, 6 h, and 7 h up to 8 h and diluted to 10 mL with phosphate-buffered saline (pH 6.8) solution and replaced with an equal volume of fresh transport medium. The absorbance was measured using a UV-VIS spectrophotometer at wavelength of 265 nm, keeping the respective blanks (phosphate-buffered saline) [6].

### 2.4. Kinetics of Intestinal Permeation Studies

In order to predict and correlate the* in vitro* intestinal permeation behavior from these famotidine-loaded microemulsions through excised goat intestine, it is necessary to fit into a suitable mathematical model. The* in vitro* famotidine permeation data from microemulsions containing famotidine through excised goat intestine were evaluated kinetically by various mathematical models like zero-order, first-order, Higuchi, and Korsmeyer-Peppas model.

### 2.5. *In Vivo* Pharmacokinetic Studies

All animal procedures were performed in accordance with protocols reviewed and approved by the Committee for the Purpose of Control and Supervision on Experimental Animals (CPCSEA). The pharmacokinetic study of the microemulsion containing famotidine was conducted in New Zealand rabbits weighing 2.5–3.0 Kg. The rabbits have been chosen as the model for study because there have been many bioavailability studies done using this animal model. The rabbits were housed individually with free access to food and water. A 12 h light/12 h dark cycle was held to keep a normal circadian rhythm in the animals.

Six rabbits were divided into three groups and fasted for 24 hours. Control batch was fed with normal saline, test batch was fed with 6 mg/kg famotidine (pure drug), and the test batch was given the formulation equivalent to 6 mg/kg of drug. Water was given ad libitum during fasting and throughout the experiment. The blood samples (approximately 300–400 *μ*L) were collected from the marginal ear vein of the rabbits using heparinized needle (20–24 size) at predetermined time intervals, specifically at 0.5, 2, 6, 8, 10, 12, and 24 hours after oral administration. The heparinized blood samples were immediately collected in centrifugation tubes (5 mL) and centrifuged at 20000 rpm at 0°C for 15 minutes. Supernatant layer of plasma was separated into another centrifugation tube and stored at −20°C until analysis [7].

### 2.6. LC-MS/MS Instrument

The 1200 Series HPLC System (Agilent Technologies, Waldbronn, Germany) was used. Mass spectrometric detection was performed on an API 3200 triple-quadrupole instrument (Applied Biosystems/MDS SCIEX, Toronto, Canada). Data processing was performed on Analyst 1.4.2 software package (SCIEX) [8].

### 2.7. Chromatographic Method Conditions

Agilent Zorbax SB-CN (50 mm × 2.1 mm I.D., 5 micron) was selected as the analytical column. The mobile phase was composed of methanol: 20 mM ammonium acetate (55 : 45, v/v). The flow rate of the mobile phase was set at 0.6 mL/min and the injection volume was 10 *μ*L. The column temperature was set at 20°C [8]. The retention time of famotidine was found to be approximately 1.37 min as shown in Table 1.

### 2.8. Sample Preparation/Extraction Procedure

An aliquot 50 *μ*L plasma was used for analysis. All samples and standards were made slightly acidic by addition of 10 *μ*L of 0.1 M aqueous ammonium acetate (pH 6) and were extracted into 3 mL of ethyl acetate. The extraction tubes were shaken at high speed for 5 min followed by centrifugation at 6000 rpm for 5 min. The organic phase was transferred to clean glass tubes and evaporated to dryness in a 45°C water bath under a nitrogen stream. The samples were reconstituted within 200 *μ*L of mobile phase and vortexed for 30 sec. After transfer into glass inserts of autosampler vials, an aliquot of 10 *μ*L of each sample was injected onto the LC-MS/MS system [9].

### 2.9. Pharmacokinetic Data Analysis

After oral administration of the microemulsion and standard drug, plasma samples were analyzed by LC-MS/MS for their famotidine content. A curve of cumulative drug absorbed Vs time curved from 0 to 24 hours was plotted to calculate the area under curve (AUC). Other pharmacokinetic parameters, that is, peak plasma level (*C*_max⁡_) and time to reach peak plasma level (*T*_max⁡_), were obtained after analysis of the individual plasma concentration-time curves. The calculations were made by computer using Win Nonlin TM Professional version 3.1 software (Pharsight Corporation, California, USA).

## 3. Results and Discussions

### 3.1. Physicochemical Evaluation of Developed Formulations

Microemulsion formulation containing olive oil was prepared using Tween-80 and PEG 400 fixed Smix ratios of 2 : 1 (Table 2). Formulation was evaluated for the various physicochemical parameters (Table 3). The narrow globule size range of 170.1 ± 1 nm and polydispersity index 0.415 ± 0.029 for F-1 indicated that the microemulsion approached a monodispersed stable system and could deliver the drug effectively owing to larger surface area. The presence of zeta potential to the tune of −6.58 ± 0.32 mV on the globules of F-1 conferred physical stability to the system. The microemulsions were expected to have good physical stability (phase separation) as zeta potential is less than −30 to −40 mV [10–12]. A percentage transmittance of 95.8% for F-1 indicated clear dispersion. The pH of the optimized famotidine microemulsion was found to be 7.1 ± 1.46, approximating the normal blood pH (7.4). It was observed that the viscosity of the microemulsion formulation generally was very low (138.5 ± 0.96 cp). This was expected, because one of the characteristics of microemulsion formulations is of lower viscosity [13–15].

### 3.2. *In Vitro* Intestinal Permeation Studies

*In vitro* drug permeation was examined through goat intestine over a period of 8 h in 0.1 (N) HCl solution at 37 ± 0.5°C for the optimized microemulsions and the corresponding pure drug. There was an increase in intestinal permeability for optimized formulation compared to pure drug due to presence of surfactants and cosurfactants combination, generally used as permeation enhancers.

**Figure 1:**
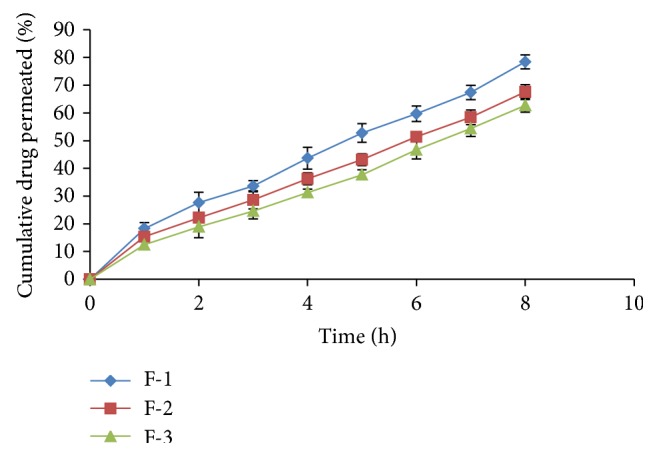
Intestinal permeability studies of formulations F-1, F-2, and F-3.

**Figure 2:**
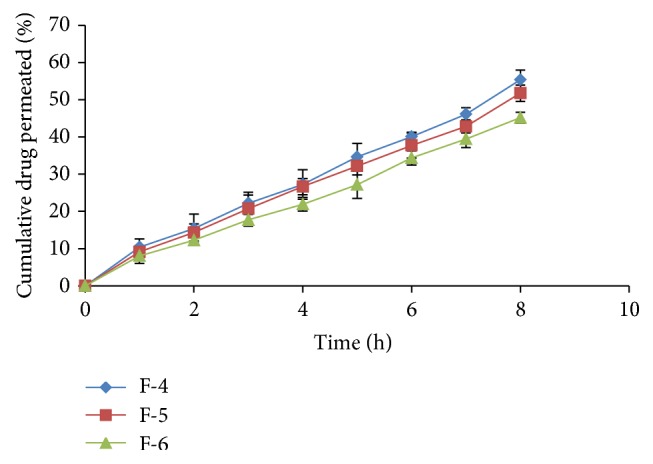
Intestinal permeability studies of formulations F-4, F-5, and F-6.

**Figure 3:**
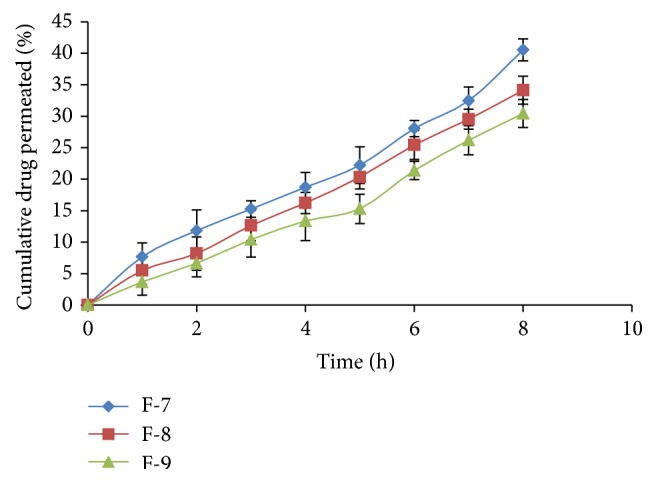
Intestinal permeability studies of formulations F-7, F-8, and F-9.

**Figure 4:**
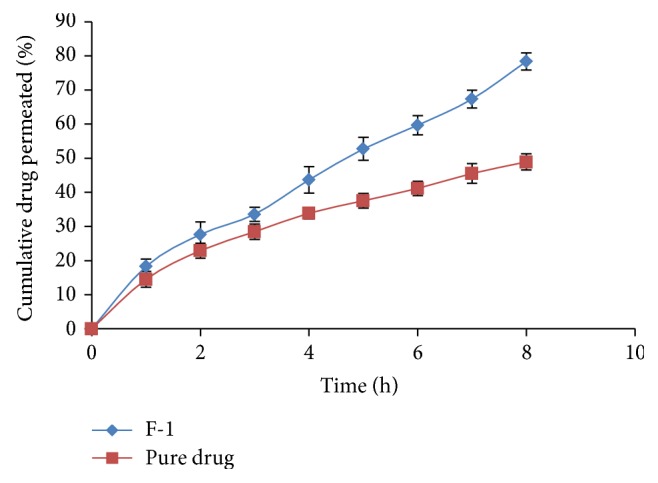
Intestinal permeability studies of pure drug and optimized formulation.

*In vitro* drug permeation in intestine after 8 h for all formulations varied from 30.42% to 78.39% (Table 4 and Figures 1, 2, and 3) and most of the formulations showed enhanced permeation compared to pure drug (48.92%) (Table 5 and Figure 4). Formulation F-1 (78.39%) shows highest permeation and F-9 (30.42%) shows lowest permeation in intestine. The maximum permeation could be due to having the lowest droplet size and lowest viscosity of all the formulations. Thus the drug diffused at a faster rate from the microemulsion system.

### 3.3. Kinetics of Intestinal Permeation Studies

In order to predict and correlate the* in vitro* intestinal permeation behavior from these famotidine-loaded microemulsions through excised goat intestine, it is necessary to fit into a suitable mathematical model. The* in vitro* famotidine permeation data from microemulsions containing famotidine through excised goat intestine were evaluated kinetically by various mathematical models like zero-order, first-order, Higuchi, and Korsmeyer-Peppas model. Based on intestinal permeation behavior of famotidine-loaded microemulsions formulations F-1 to F-7 have been selected for kinetic evaluation. The results of the curve fitting into these above-mentioned mathematical models indicate the* in vitro* intestinal permeation behavior of famotidine-loaded microemulsions (F-1 to F-7) shown in Table 6. When respective correlation coefficients were compared, F-1 followed the zero-order model (*R* ^2^ = 0.986), whereas F-2, F-3, and F-4 formulations followed Korsmeyer-Peppas model (*R* ^2^ = 0.990, 0.998, and 0.989), and formulation F-5, F-6, and F-7 followed the first-order release (*R* ^2^ = 0.995, 0.994, and 0.998) over a period of 8 hours. The determined values of diffusion exponent (*n*) ranged between 0.610 and 1.329, indicating that the intestinal drug permeation from these famotidine-loaded microemulsions followed the Supercase II transport.

### 3.4. *In Vivo* Pharmacokinetic Studies

**Figure 5:**
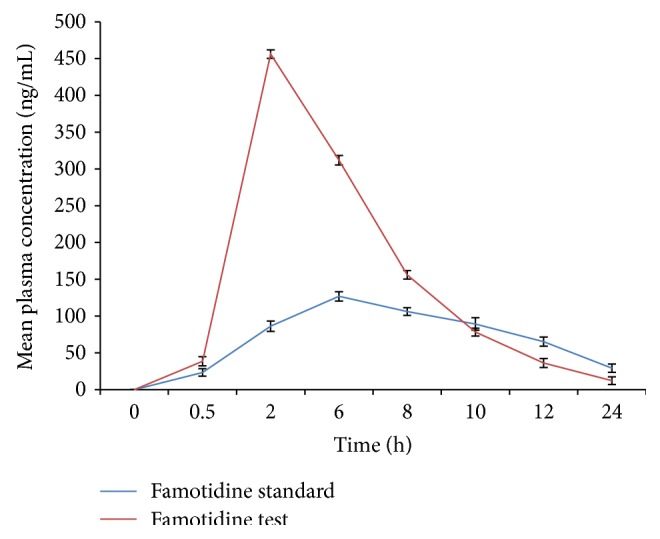
Comparison of pharmacokinetic profiles of standard drug and oral microemulsion of famotidine (F-1).

The* in vivo* study was performed to quantify famotidine, after oral administration of formulation containing drug. The plasma concentration time profiles of the drug in male New Zealand albino rabbits following oral administration of the microemulsion formulation (F-1) and standard drug were compared.  Figure 5 shows mean plasma concentration-time curve of famotidine after a single oral administration of standard drug and test formulation. The oral pharmacokinetic parameters are presented in Tables 7 and 8. Famotidine microemulsions (F-1) demonstrated a longer *T*_max⁡_ (6 h) compared with standard drugs (2 h) and sustained the release of drugs over 24 h because the drug needs to be released out from the oil phase thereby resulting in a delayed *T*_max⁡_ [16]. After oral administration, F-1 exhibited the higher absorption and *C*_max⁡_ achieved from the optimized famotidine test formulation (456.20 ng·h/mL) represents greater improvement than the standard drug (126.80 ng·h/mL). The drug content of the test formulation was significantly higher at all time periods after administration than that of the standard formulation. It was found that AUC_0–24_ h obtained from the optimized famotidine test formulation (3023.5 ng·h/mL) was significantly higher than the standard famotidine (1663.3 ng·h/mL). Area under the curve (AUC) for microemulsion showed almost a 1.8-fold increment from AUC generated after administering standard famotidine indicating a significant enhancement of famotidine bioavailability when given orally as microemulsions [17]. The obtained result confirms the superior bioavailability of test formulation than the standard drug. The significant differences of the factors leading drug absorption* in vivo* between the microemulsion preparations and standard drugs were probably attributed to the following.

Famotidine belongs to BCS class-III drug and the oral absorption as well as the bioavailability of both drugs is mainly limited due to low intestinal permeability. The surfactant and cosurfactant (Tween-80 and PEG 400) may have contributed to an increase in the permeability of the intestinal membrane or improved the affinity between lipid particles and the intestinal membrane. Further, due to small particle size, microemulsions may adhere to the gut membrane or enter the inter-villar spaces thus extending gastrointestinal residence time in the gastrointestinal tract [18]. Moreover, microsized preparation ensures greater surface area and also the presence of Tween-80 as a surfactant in the microemulsion formulation might modulate the intestinal membrane permeability through apically polarized efflux system leading to enhanced oral bioavailability [19].

## 4. Conclusion

The results from these studies demonstrated that microemulsion is a viable approach for developing a liquid dosage form of famotidine with enhanced intestinal permeability as well as bioavailability. Enhancing permeability correlates with improved pharmacokinetic profile. The pharmacokinetic studies reveal that the oral administration of famotidine microemulsion sustained the release of drugs over 24 h. As a consequence of this, decrease in the dose and frequency of administration for drugs is possible to achieve the desired therapeutic activity. This study proved the utilization of microemulsion as a carrier for oral delivery of famotidine.

## Figures and Tables

**Table 1:** Chromatographic condition for pharmacokinetic study.

	Chromatographic conditions
LC-MS/MS	API 3200, MDS SCIEX
Ionization source	ESI
Column	Agilent Zorbax SB-CN (50 mm × 2.1 mm I.D., 5 micron)
Mobile phase	Methanol: 20 mM ammonium acetate (55 : 45, v/v)
Flow rate	Isocratic flow rate of 0.6 mL/min
Detection	MS/MS
Extraction solvent	3 mL of ethyl acetate
Injection volume	10 *μ*L
Retention time of famotidine	1.37 min
Linearity range	5 to 5000 ng/mL
Rabbit plasma volume used	50 *μ*L

**Table 2:** Composition of optimized famotidine microemulsion.

Ingredients (by wt)	Famotidine microemulsion (F-1)
Famotidine (mg/mL)	40
Olive oil	7.14
Smix (Tween-80 : PEG 400)	64.29
Water	28.57

**Table 3:** Evaluation parameters of optimized microemulsion (*n* = 3).

Formulation	pH	Globule size (nm) ± SEM	PDI ± SEM	Zeta potential (mV) ± SEM	Viscosity (cp)	Percentage transmittance
F-1	7.12 ± 1.46	170.1 ± 1	0.415 ± 0.029	−6.58 ± 0.32	138.5 ± 0.96	95.8

**Table 4:** * In vitro* intestinal permeation studies for famotidine microemulsions.

F. code	% cumulative drug permeated								
	0 h	1 h	2 h	3 h	4 h	5 h	6 h	7 h	8 h
F-1	0	18.29 ± 2.16	27.64 ± 3.71	33.58 ± 2.06	43.67 ± 3.91	52.74 ± 3.37	59.68 ± 2.80	67.34 ± 2.60	78.39 ± 2.52
F-2	0	15.34 ± 1.76	22.16 ± 1.32	28.64 ± 3.22	36.23 ± 2.17	43.18 ± 2.12	51.37 ± 1.47	58.43 ± 2.66	67.54 ± 2.66
F-3	0	12.47 ± 0.43	18.92 ± 3.93	24.61 ± 2.84	31.28 ± 1.22	37.72 ± 1.80	46.68 ± 3.35	54.35 ± 2.82	62.73 ± 2.49
F-4	0	10.41 ± 2.20	15.36 ± 3.89	22.18 ± 2.99	27.25 ± 3.96	34.63 ± 3.61	40.09 ± 1.17	46.11 ± 1.76	55.37 ± 2.57
F-5	0	9.12 ± 1.77	14.32 ± 2.30	20.73 ± 3.70	26.68 ± 2.18	32.18 ± 2.37	37.72 ± 3.39	42.84 ± 1.72	51.77 ± 2.19
F-6	0	8.04 ± 2.04	12.23 ± 1.24	17.71 ± 1.62	21.92 ± 1.82	27.20 ± 3.70	34.37 ± 1.909	39.48 ± 2.34	45.19 ± 1.45
F-7	0	7.67 ± 2.21	11.82 ± 3.29	15.25 ± 1.31	18.71 ± 2.34	22.21 ± 2.91	28.04 ± 1.28	32.46 ± 2.17	40.53 ± 1.75
F-8	0	5.49 ± 2.24	8.18 ± 2.65	12.64 ± 2.44	16.21 ± 1.69	20.32 ± 1.89	25.44 ± 2.31	29.52 ± 1.58	34.12 ± 2.23
F-9	0	3.68 ± 2.09	6.64 ± 2.15	10.39 ± 2.77	13.36 ± 3.12	15.29 ± 2.33	21.38 ± 1.45	26.17 ± 2.31	30.42 ± 2.22

Data are presented as mean ± S.D.

**Table 5:** * In vitro* intestinal permeation studies for pure famotidine.

F. code	% cumulative drug permeated								
	0 h	1 h	2 h	3 h	4 h	5 h	6 h	7 h	8 h
Pure drug (famotidine)	0	14.51 ± 2.31	22.92 ± 2.19	28.45 ± 2.26	33.89 ± 1.68	37.53 ± 2.16	41.15 ± 2.10	45.53 ± 2.87	48.92 ± 2.36

**Table 6:** Famotidine microemulsions intestinal permeation kinetics data.

F. code	Zero-order *R* ^2^	First-order *R* ^2^	Higuchi kinetics *R* ^2^	Korsmeyer-Peppas *R* ^2^	Diffusion exponent (*n*)
F-1	0.986	0.984	0.774	0.977	1.283
F-2	0.979	0.983	0.777	0.990	1.329
F-3	0.996	0.998	0.926	0.998	0.849
F-4	0.877	0.947	0.993	0.989	0.610
F-5	0.992	0.995	0.929	0.986	0.784
F-6	0.993	0.994	0.928	0.991	0.812
F-7	0.994	0.998	0.925	0.988	0.752

**Table 7:** Results of pharmacokinetics study on standard famotidine and test formulation.

Time (h)	Concentration (ng/mL)	
	Famotidine standard	Famotidine test (F-1)
0.00	0.000	0.000
0.50	23.40 ± 5.12	38.60 ± 6.16
2.00	86.20 ± 7.08	456.20 ± 5.68
6.00	126.80 ± 6.17	312.00 ± 6.54
8.00	106.20 ± 5.32	156.00 ± 5.73
10.00	89.20 ± 8.40	78.20 ± 5.49
12.00	65.20 ± 6.26	36.10 ± 6.10
24.00	29.20 ± 5.61	12.20 ± 5.34

Data are presented as mean ± S.D.

**Table 8:** Pharmacokinetic data of standard famotidine and test formulation.

Pharmacokinetic parameters		Famotidine standard	Famotidine test (F-1)
AUC_(0–24)_	(ng·h/mL)	1663.3	3023.5
*C*_max⁡_	(ng/mL)	126.80	456.20
*T*_max⁡_	(h)	2.000	6.000

Data are presented as mean ± S.D. of four animals.
